# The effects of transcranial direct current stimulation (tDCS) on subjective, autonomic and neuropsychological response in the 7.5% CO_2_ experimental model of anxiety

**DOI:** 10.1177/02698811261420089

**Published:** 2026-03-11

**Authors:** Joanna Astrid Miler, Daniel Meron, David S. Baldwin, Matthew Garner

**Affiliations:** 1 Department of Psychology, School of Social Sciences, Edinburgh Napier University, UK; 2 School of Psychology, Faculty of Environmental and Life Sciences, University of Southampton, UK; 3 Clinical and Experimental Sciences, Faculty of Medicine, University of Southampton, UK; 4 University Department of Psychiatry and Mental Health, University of Cape Town, South Africa

**Keywords:** noninvasive brain stimulation, direct current stimulation, carbon dioxide challenge, anxiety, cognitive bias

## Abstract

**Background:**

Anxiety is characterized by hypervigilance, distractibility and selective processing of negative information. There is growing evidence that prefrontal function underlies biases in threat processing and attention control in anxiety. We examined the effect of 20 min of 2 mA dorsolateral prefrontal cortex transcranial direct current stimulation (tDCS) (bipolar-balanced montage) on subjective anxiety, autonomic arousal and threat processing in the 7.5% CO_2_ experimental medicine model of anxiety.

**Methods:**

A between-subjects healthy volunteer double-blind randomized design compared 2 mA tDCS stimulation of the PFC versus sham tDCS on subjective anxiety, autonomic arousal and antisaccade performance during 7.5% CO_2_ challenge.

**Results:**

tDCS did not moderate subjective and autonomic response to CO_2_ challenge. tDCS reduced erroneous eye movements toward threat images relative to neutral images.

**Conclusion:**

Twenty minutes of active 2 mA tDCS over the left dorsolateral prefrontal cortex may reduce threat processing biases during experimentally induced anxiety and could help target early positive changes in emotion processing.

## Highlights

- Experimental medicine models of anxiety support the evaluation of novel therapeutics such as noninvasive brain stimulation.
- We evaluated prefrontal transcranial direct current stimulation in the CO_2_ model of anxiety.
- CO_2_ inhalation increased anxiety, heart rate and blood pressure, similarly in active and sham tDCS groups.
- tDCS reduced erroneous eye movements to threat versus neutral stimuli during the CO_2_ challenge.
- Follow-up studies may test whether early tDCS-induced changes in threat processing precede improvement in anxiety symptoms.

## Introduction

Anxiety is characterized by hypervigilance, distractibility and selective processing of negative information (Penninx et al., 2021). Individuals with elevated levels of state or trait anxiety preferentially allocate attention toward threatening stimuli compared to neutral stimuli, and this “bias” is greater than that observed in non-anxious individuals or under low anxiety conditions (Bar-Haim et al., 2007; Boschloo et al., 2019; Dudeney et al., 2015). Heightened states of anxious apprehension and persistent uncontrollable worry may result from dysfunction in amygdala-prefrontal circuits, including the prefrontal cortex (PFC) that controls maladaptive attentional biases to anxiogenic threat stimuli (Akiki et al., 2025; Carlisi and Robinson, 2018; Grupe and Nitschke, 2013).

tDCS is a noninvasive brain stimulation method, which alters cortical tissue excitability by applying a weak (0.5–2 mA) constant direct current via scalp electrodes to the cortical region of interest (Nitsche and Paulus, 2000). Anodal stimulation facilitates cortical activity, whereas cathodal tDCS inhibits activity. In contrast to transcranial magnetic stimulation (TMS), tDCS does not directly trigger action potentials in neuronal cells, but instead changes overall tissue excitability (Priori et al., 2009). Moreover, as opposed to TMS, tDCS can modulate the cortical regions during the completion of a task and is easier to use in double-blind sham-controlled studies. Recent meta-analyses suggest noninvasive brain stimulation can reduce clinical anxiety, though therapeutic effects are greater for TMS and comparatively small and not consistently observed with tDCS (Qi et al., 2024), consistent with findings across mental disorders (review by Hyde et al. (2022). Evidence from case study/series suggests excitatory stimulation of left prefrontal or inhibitory stimulation of right prefrontal areas may be optimal (Sagliano et al., 2019; Vicario et al., 2019).

Recent studies have examined the effects of tDCS on attention to emotional information. Twenty minutes of active dorsolateral prefrontal cortex (DLPFC) tDCS with a bipolar-balanced montage can reduce vigilance to threat (Ironside et al., 2016), and 17 minutes of 1 mA monopolar anodal stimulation of left PFC can enhance attention training procedures that direct attention away from threat stimuli (Clarke et al., 2014). Moreover, 20 min 2 mA anodal stimulation over the right DLPFC impairs attentional disengagement from both positive and negative faces in an eye-tracking attention task in healthy volunteers, suggesting a causal role of right DLPFC activity in the generation of attentional impairments implicated in emotional disturbances such as depression and anxiety (Sanchez et al., 2016). 20 minutes of active 2 mA tDCS over left DLPFC is associated with greater executive attention control in healthy humans (Miler et al., 2018); and bipolar tDCS of the DLPFC can reduce amygdala fear signaling and increase frontoparietal attentional control, subsequently reducing attentional capture by threat in high trait anxious individuals (Ironside et al., 2019). There is also evidence that 25 minutes of active anodal 2 mA tDCS over the left DLPFC can reduce attentional bias in patients with social anxiety disorder (Heeren et al., 2017). More recently, tDCS may help dissociate neural mechanisms that underlie maladaptive biases in attention versus interpretation in anxiety (Nejati et al., 2021).

Experimental medicine models in healthy volunteers provide efficient and cost-effective pre-clinical evaluations of novel treatment methods (Grillon et al., 2019). In the 7.5% CO_2_ inhalational model of anxiety, healthy volunteers inhale air “enriched’ with 7.5% CO_2_ (“CO_2_ challenge”). In healthy humans, this model mimics the subjective, autonomic and neuropsychological features of GAD (Bailey et al., 2007; Garner et al., 2011). CO_2_ challenge increases functional connectivity in anxiety-related regions, including medial frontal cortex, amygdala and brainstem (Goossens et al., 2014; Graham et al., 2024), and functional connectivity in prefrontal-amygdala networks (at baseline) predicts anxiety during CO_2_ challenge (Huneke et al., 2020). CO_2_ challenge can induce hypervigilance toward and deficient inhibition of visual threat stimuli, e.g. in eye-tracking antisaccade tasks (Garner et al., 2011), consistent with evidence that patients with GAD orient more readily toward threat stimuli in other eye-tracking paradigms (Ashwin et al., 2012). CO_2_ challenge increases hypervigilance in spatial and temporal domains (Garner et al., 2012) and can impair fronto-executive functions of cognitive flexibility and working memory using the Cambridge Neuropsychological Test Automated Battery (CANTAB) Intra-Extra Dimensional Set Shift, Affective Go/No-go and Spatial Working Memory tasks (Savulich et al., 2019). Moreover, anxiety induced in the CO_2_ model is sensitive to anxiolytic pharmacological and psychological treatments (Ainsworth et al., 2015; Bailey et al., 2007).

We examined the effect of 20 minutes of 2 mA prefrontal DLPFC tDCS on CO_2_-induced anxiety, autonomic arousal and attention to threat in healthy participants. In addition to measures of subjective anxiety (self-report) and autonomic arousal (blood pressure, heart rate), we measured attention to threat and inhibitory control in an antisaccade task (Garner et al., 2011). In this task, top-down attention control is required to suppress (inhibit) reﬂexive saccades (eye movements) toward an abrupt peripheral visual stimulus and instead generate a voluntary saccade in the opposite location (antisaccade). The antisaccade task provides two distinct performance measures: (i) performance effectiveness—that is the proportion of trials on which participants are unable to successfully make antisaccades (i.e., error rate) and (ii) processing efﬁciency—that is the time required to successfully program and make correct saccades (Kristjansson, 2007). Anxiety is known to impair both antisaccade accuracy and latency, with antisaccades having longer latencies and higher error rates in anxious individuals (Ansari and Derakshan, 2011; Derakshan et al., 2009; Eysenck et al., 2023). Moreover, evidence from neuroimaging studies suggests that DLPFC activation underlies antisaccade performance, for example (Ettinger et al., 2008).

The antisaccade task provides a reliable and sensitive measure of processes involved in resolving the conflict between volitional and reflexive behavioral responses and is a comparatively simple behavioral measure (see Hutton and Ettinger, 2006). Only a handful of studies using the antisaccade task in tDCS neuromodulation studies have been reported. These indicate that bilateral tDCS of the frontal eye fields (FEF) can modulate the function of FEF to suppress reflexive saccades to the contralateral visual cue (Kanai et al., 2012); and can lead to a significant reduction in antisaccade errors in schizophrenia (Subramaniam et al., 2015).

Following recommendations from meta-analysis (Horvath et al., 2015), we used a robust physical and mental health screening procedure to match groups; examined subjective and autonomic state-dependent measures of mood/arousal at baseline and post-stimulation; and examined retrospective expectancies about stimulation condition that might have affected predicted effects of stimulation. Informed by previous literature utilizing the 7.5% CO_2_ model of anxiety, we predicted a robust effect of the CO_2_ inhalation on subjective anxiety and autonomic arousal (relative to baseline and post-tDCS) and examined the effect of tDCS on subjective and autonomic response. We predicted that a 20-minute session of active prefrontal tDCS would improve attention control and reduce erroneous eye movements to threat in an antisaccade task during CO_2_-induced anxiety compared to sham tDCS.

## Materials and methods

### Participants

**Figure 1.**
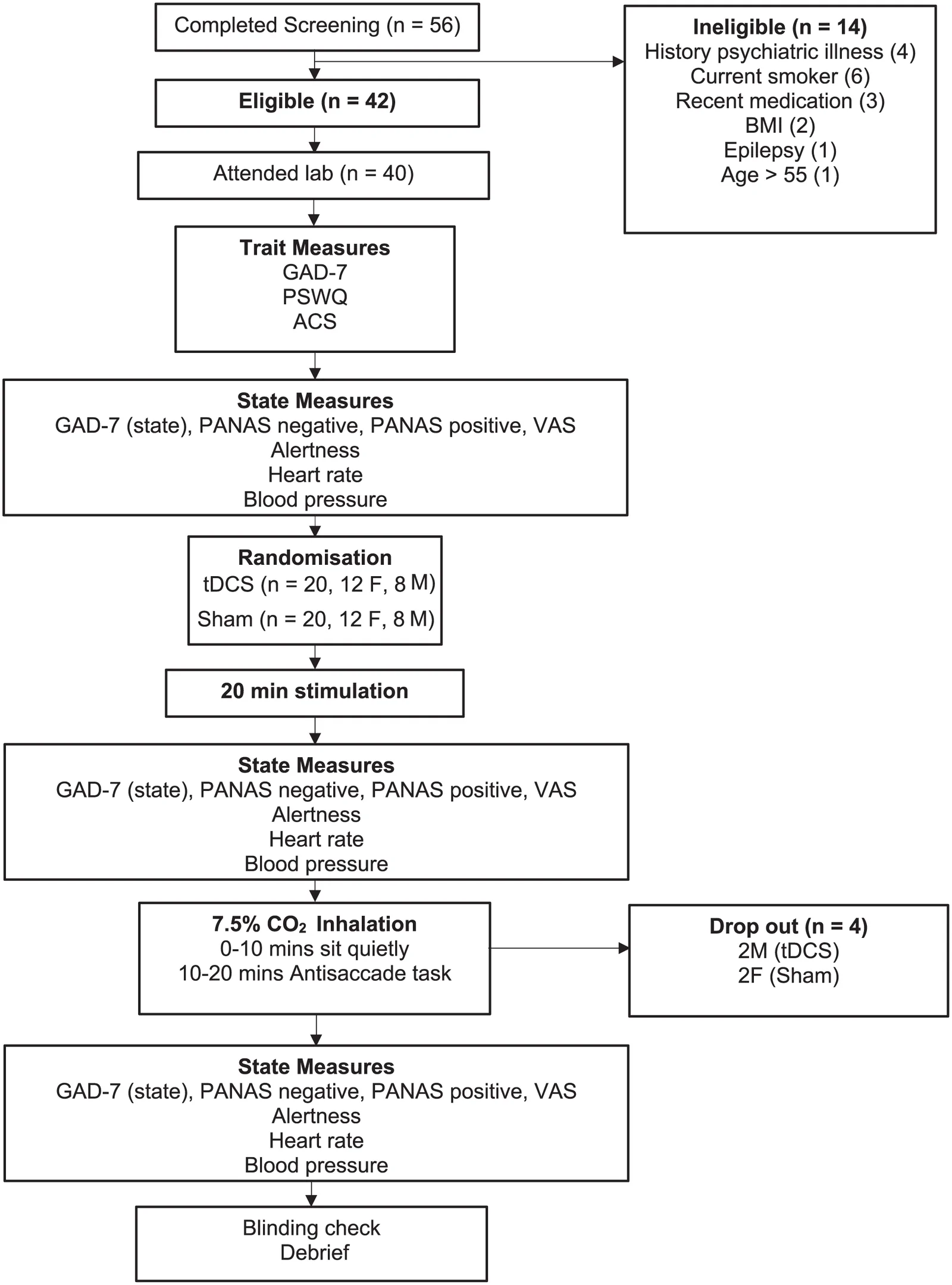
Summary of study protocol.

We recruited *N* = 42 participants (21 per group); with α = 0.05, we had 80% power to detect large effects of *d* ⩾ 0.80. We recruited healthy volunteers through online adverts. Participants underwent a telephone health screen prior to the testing session, and a mental health screen on the day of the study session, using a structured diagnostic interview [Mini International Neuropsychiatric Interview] (Sheehan et al., 1998). A physical health checklist screened participants for current and lifetime physical illness exclusion criteria. Fifty six respondents were screened. Eligible participants were required to be aged 18–55 years. All participants were right-handed. Exclusion criteria included metal or electronic implants, epilepsy, recent medication (past 8 weeks bar topical treatment, paracetamol, oral, injectable, or skin patch contraception), pregnancy, elevated blood pressure (>140/90 mm Hg), cardiovascular disease, lifetime history of psychiatric illness/alcohol/drug dependence, current smoker, body mass index < 18 or ⩾28 kg/m^2^, and recent use of alcohol (conﬁrmed by breath test). Fourteen respondents were ineligible (see Figure 1). Forty two eligible participants were invited to participate. Forty participants attended the lab, of which four withdrew during CO_2_ inhalation (2 per stimulation group). Consequently, we report data from 36 participants—18 per group (see Table 1). The research was approved by the Ethics and Research Governance Committee, School of Psychology, University of Southampton and conducted in experimental laboratories with controlled lighting and temperature. All procedures complied with the Helsinki Declaration of 1975, as revised in 2008. Participants provided informed consent, were debriefed upon completion, and compensated with either ‘study participation credits’ (if university students) or with ﬁnancial compensation (£6/hour).

**Table 1.** Sample characteristics (trait measures).

		Active tDCS		Sham tDCS	
		*n* = 18		*n* = 18	
		(12 females)		(10 females)	
		Mean age = 21.44 yrs (SD = 2.87)		Mean age = 21.29 yrs (SD = 3.16)	
Trait measures	Time	*M*	SD	*M*	SD
GAD 7 (Anxiety)	Pre-session	21.28	10.56	18.09	10.35
PSWQ	Pre-session	38.22	13.44	37.06	12.05
ACS	Pre-session	50.76	7.08	49.28	9.52

ACS: Attention Control Scale; GAD: general anxiety disorder; PSWQ: Penn State Worry Questionnaire; tDCS: transcranial direct current stimulation; yrs: years.

### Protocol

See Figure 1 for the study overview. Sample characteristics (trait measures) of GAD-7 (Kroenke et al., 2007), the Anxiety Sensitivity Index (ASI) (Reiss et al., 1986), the Attention Control Scale (ACS) (Derryberry and Reed, 2002), and the Penn State Worry Questionnaire (PSWQ) (Meyer et al., 1990) were taken at the start of the session. The following primary outcome measures were taken at baseline, immediately post-intervention and immediately after 7.5% CO_2_ challenge: heart rate beats per minute(BPM), diastolic and systolic blood pressure (Omron-M6 arm-cuff monitor, Medisave, UK), subjective alertness (visual analogue VAS), as well as the Positive and Negative Affect Scale (PANAS; (Watson et al., 1988)), and a modified version of GAD-7.

The tDCS intervention was a 20 minutes double-blind 2 mA stimulation with a bipolar-balanced montage (anode centered over left DLPFC, F3,^1^ cathode centered over right DLPFC, F4) or nonactive sham tDCS (HDCkit, Magstim, UK). We utilized a comparatively high current intensity of 2 mA with 4 × 4 cm electrodes encased in saline-soaked sponge pads to achieve a current density 50.125 mA/cm^2^, akin to (Heeren et al., 2015; Ironside et al., 2019). In the active condition, the stimulator supplied the 2 mA current for 20 minutes. In the sham condition, 2 mA stimulation was ramped up and delivered for the ﬁrst 15 seconds only. The participant was instructed to remain seated, relaxed, and refrain from any motor activity for the duration of the stimulation. Immediately after the intervention physiological and subjective measures were collected. Participants were then prepared for the 7.5% CO_2_ challenge, during which they inhaled air enriched with 7.5% CO_2_ (21% O_2_, balance N_2_) for 20 minutes through an oral-nasal face mask. Midway through the inhalation participants began the antisaccade task. The antisaccade task lasted approximately 8 minutes and all participants completed the task before the end of the 20 minutes inhalation. The test session lasted approximately 3 hours. Participants were debriefed and contacted 24 hours later to discuss any further queries and register any side effects; none were reported.

### Antisaccade task

In the emotional version of the antisaccade eye-movement task, participants were instructed to look toward (prosaccade) or look away from (antisaccade) eight negative and eight neutral pictures selected from the standardized International Affective Picture Set ([CSEA-NIMH], 1999). This task provides measures of attention control (i.e., ability to inhibit eye movements to pictures on antisaccade trials) and selective attention (i.e., speed and likelihood of looking toward negative relative to neutral stimuli). Participants completed 96 trials presented in random order (24 trials per saccade-type × picture valence condition). Trials were counter-balanced for stimulus location (thus there were 12 trials per trial type × location × valence condition). On each trial, a word (either ‘TOWARDS’ or ‘AWAY’) was presented at central fixation for 2000 ms and instructed participants to look toward (prosaccade) or away from (antisaccade) the picture stimulus (i.e., to shift their gaze to the opposite side of the screen). At 200 ms following word offset, the picture stimulus was presented for 600 ms (6° to the left or right of central fixation). There were 96 randomized experimental trials, prior to which participants completed eight practice trials in which they completed pro- and anti-saccades to a peripheral yellow rectangle. Participants were asked to classify the direction of a small arrow (↑ or ↓) presented at 50 ms following picture offset (arrow-picture location congruent on 50% of trials per trial type) to increase task demand on each trial. The task was presented using Inquisit 2 computer software. Consistent with (Garner et al., 2011), horizontal eye movements were measured by electro-oculography and sampled at 1000 Hz (MP150-amplifier and AcqKnowledge 3.8.1 software, Biopac systems, Goleta, CA).

### Data analysis

Saccade direction and latency were scored manually and blind to trial type and inhalation condition using AcqKnowledge software. In accordance with previous literature (e.g., Garner et al., 2011) saccades with a latency less than 100 ms (i.e., anticipatory eye movements) as well as saccades which subtended less than six horizontal degrees (i.e., did not terminate in the location/mirror location of the stimulus) were removed from the analyses (with no difference between groups, Fs < 1). The mean correct values for accuracy from left and right visual fields were combined, and the mean value was used (x/12).

We screened for variables that moderated the effect of CO_2_ on outcome measures. Response to CO_2_ was higher in female versus male participants—consequently, we entered gender as a covariate in all analyses. Neither age nor trait measures (self-reported anxiety, worry, attention control) covaried with CO_2_ effects. Separate 2(Group: active versus sham) × 3(Time: baseline versus post-stimulation versus post-CO_2_) mixed design analyses of covariance (ANCOVA) were tested for group differences in anxiety, mood, heart rate (HR), and blood pressure over time.

Both task accuracy and eye-movement latency data were entered into two separate omnibus 2 (Group: active versus sham) by 2 (trial type: pro- versus anti- saccade) by 2 (valence: threat versus neutral) ANCOVAs. The omnibus analyses tested for group differences in task accuracy and latency (i.e., main effect of Group) and interactions that would reﬂect an effect of tDCS stimulation on task performance. All analyses were performed using SPSS software v23 (IBM Corporation (Armonk, NY, USA)) and JASP v0.19.1. (JASP Services BV, Amsterdam, the Netherlands).

## Results

### Effects of 2 mA anodal tDCS of the left DLPFC on subjective anxiety, autonomic arousal, and response to the CO_2_ challenge

Active and sham tDCS groups did not significantly differ on pre-existing anxiety and worry (GAD-7; PSWQ), attention control (ACS), nor state mood (PANAS), state anxiety and alertness (VAS), heart rate, nor blood pressure (see Tables 1 and 2, *t*s (34) < 0.92, *p*s > .37).

**Table 2.** Subjective anxiety, mood and autonomic arousal at session baseline (pre-stimulation), post-stimulation and post-CO_2_ challenge.

		Active tDCS		Sham tDCS		Time F(2,66)	Group F(1,33)	Time × Group F(2,66)
State measure	Time	*M*	SD	*M*	SD			
GAD 7 (Anxiety)	Baseline	13.64	12.43	14.32	9.28	7.261**, *np*^2^ = 0.180	0.033, ns, *np*^2^ < 0.001	0.934, ns, *np*^2^ < 0.001
	Post-tDCS	18.33	18.24	11.90	10.43			
	Post-CO_2_	41.07	25.69	41.92	24.30			
PANAS-Positive	Baseline	31.17	7.02	31.61	7.41	8.256**, *np*^2^ = 0.210	0.011, ns, *np*^2^ < 0.001	0.664, ns, *np*^2^ = 0.02
	Post-tDCS	29.12	8.02	28.67	8.93			
	Post-CO_2_	23.41	8.20	22.28	7.65			
PANAS-Negative	Baseline	12.44	4.26	12.17	2.30	5.834**, *np*^2^ = 0.154	0.843, ns , *np*^2^ = 0.026	1.558, ns, *np*^2^ = 0.046
	Post-tDCS	11.78	2.76	11.50	1.98			
	Post-CO_2_	17.47	7.30	20.28	7.39			
Alertness	Baseline	49.94	20.26	58.00	25.70	4.877*, *np*^2^ = 0.132	0.787, ns, *np*^2^ = 0.024	0.508, ns, *np*^2^ = 0.053
	Post-tDCS	46.97	21.70	49.14	30.24			
	Post-CO_2_	65.19	36.73	71.56	27.70			
Heart rate (BPM)	Baseline	72.22	12.62	72.78	9.91	4.107**, *np*^2^ = 0.111	.014, ns, *np*^2^ < 0.001	0.452, ns, *np*^2^ = 0.014
	Post-tDCS	69.00	20.33	71.11	9.20			
	Post-CO_2_	80.20	15.91	77.72	14.54			
SBP (systolic blood pressure)	Baseline	117.94	12.83	122.06	11.96	4.7837*, *np*^2^ = 0.130	0.680, ns, *np*^2^ = 0.021	0.478, ns, *np*^2^ = 0.015
	Post-tDCS	108.11	29.93	116.67	12.24			
	Post-CO_2_	121.94	13.54	128.94	15.05			
DBP (diastolic blood pressure)	Baseline	70.94	17.06	71.17	7.06	0.105, ns, *np*^2^ = 0.003	0.03, ns, *np*^2^ = 0.01	0.037, ns, *np*^2^ = 0.001
	Post-tDCS	61.00	22.02	69.94	10.11			
	Post-CO_2_	71.94	9.97	73.00	8.13			

* *p* < 0.05. **p < 0.01.

**Figure 2.**
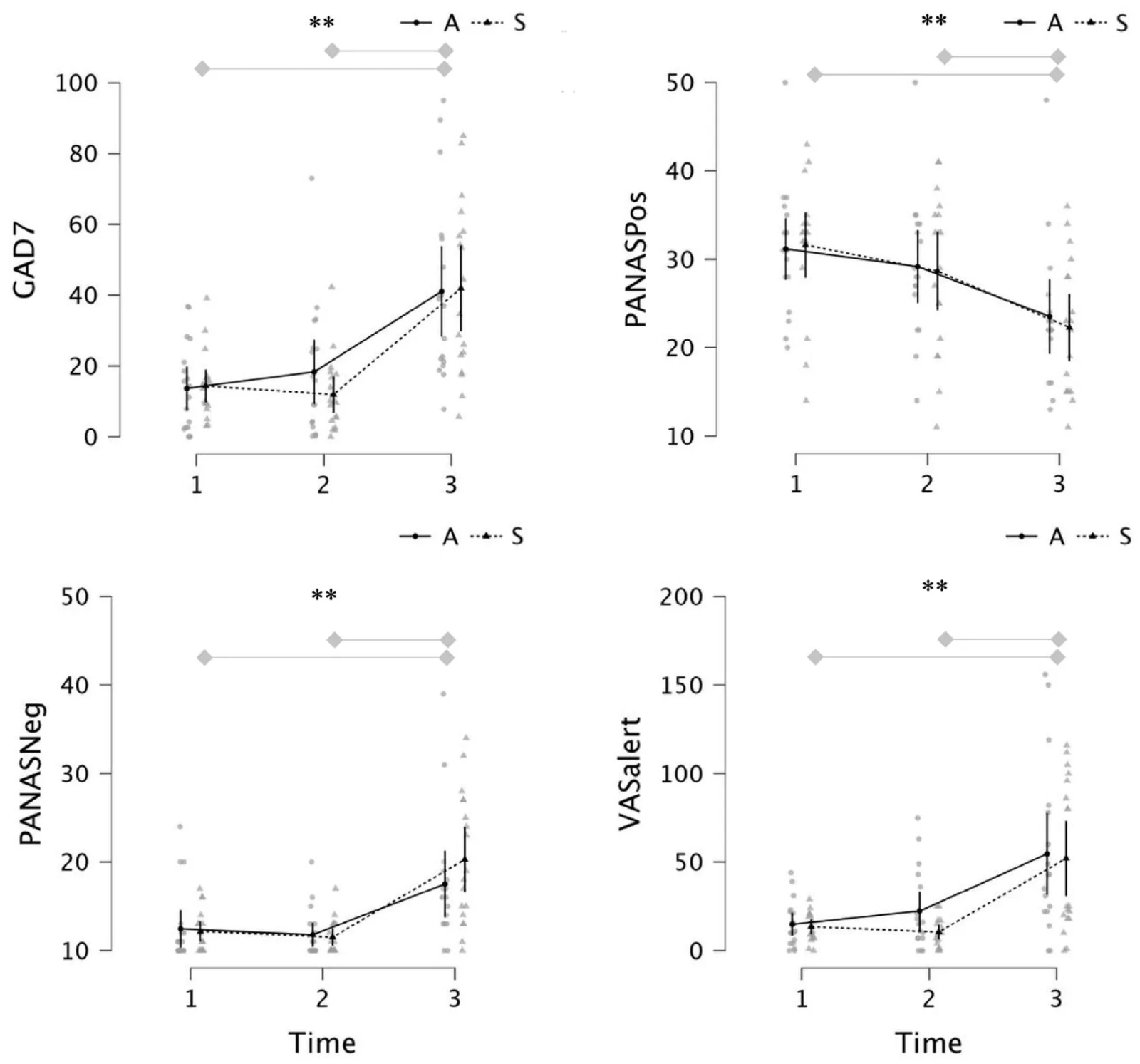
Anxiety (top left), positive affect (top right), negative affect (bottom left) and alertness (bottom right) at baseline (1), post-stimulation (2) and post CO_2_ inhalation (3) in “Active” and “Sham” groups. 95% confidence intervals. *p_Bonf_ = <0.05. **p_Bonf_ = <0.01.

As expected, CO_2_-inhalation significantly increased self-report anxiety, blood pressure and heart rate across participants, and in each group (see Figure 2 and Table 2 for descriptive and inferential statistics). ANCOVA revealed main effects of Time for self-report anxiety, positive and negative affect and alertness, characterized by large increases in anxiety, negative affect, and alertness and decreases in positive affect, following CO_2_-challenge (relative to baseline and post-stimulation (see Figure 2 for Bonferroni post-hoc comparisons). We did not find evidence of time × Group effects on anxiety, affect, or alertness (see Table 2). Taken together, the findings suggest that a single session of active anodal tDCS does not alter reported levels of anxiety, affect and alertness, nor the anxiogenic response to CO_2_-challenge in healthy young volunteers.

**Figure 3.**
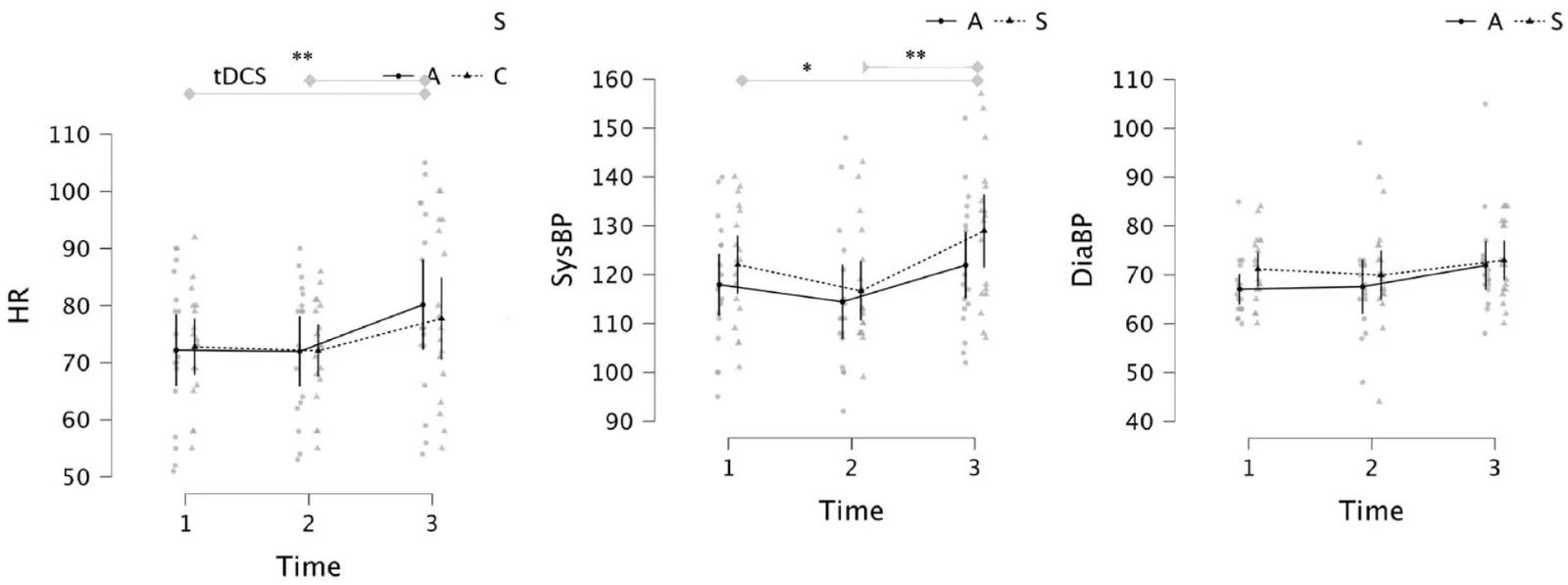
Heart rate (left), systolic blood pressure (middle) and diastolic blood pressure (right) at baseline (1), post-stimulation (2) and post CO_2_ inhalation (3) in “Active” and “Sham” groups. 95% confidence intervals. *p_Bonf_ = <0.05. **p_Bonf_ = <0.01.

ANCOVA revealed main effects of Time on heart rate and systolic blood pressure characterized by large increases in heart rate and systolic blood pressure after CO_2_ inhalation relative to baseline and post-stimulation (see Figure 3 and Table 2 for descriptive and inferential statistics). Diastolic blood pressure did not vary over time. We did not find evidence of time × Group effects on heart rate nor blood pressure (see Table 2). In summary, inhalation of 7.5% CO_2_ produced robust increases in autonomic arousal, however the effect of CO_2_ on arousal was unaffected by tDCS group. Consequently, any observed differences in antisaccade performance are unlikely to be due to group differences at baseline, nor effects of tDCS on subjective/autonomic outcomes after stimulation and after CO_2_ inhalation.

### Antisaccade task

#### Accuracy

An omnibus group(2) × trial-type(2) × valence(2) ANCOVA revealed a main effect of trial type (*F*(1, 31) = 81.613, *p* < .001, η*p*^2^ = 0.725), characterized by reduced eye-movement errors (proportion) on prosaccade (*m* = 0.04) compared to antisaccade trials (*m* = 0.59; see Table 3). A valence × group interaction (*F*(1,31) = 4.78, *p* = 0.037, η*p*^2^ = 0.134) was characterized by fewer errors on negative versus neutral trials in the active group only, with no difference between neutral and negative trials in the sham group.

**Table 3.** Error rates (proportion of trials) by trial type and valence in the active and sham tDCS conditions.

Saccade type	Active tDCS		Sham tDCS	
	*M*	SD	*M*	SD
Prosaccade Negative	0.03	0.06	0.04	0.04
Prosaccade Neutral	0.04	0.05	0.04	0.06
Antisaccade Negative	0.59^a^	0.22	0.63	0.18
Antisaccade Neutral	0.66^b^	0.21	0.61	0.21

Values with different superscripts (*a,b*) differ *p* < 0.05.

tDCS: transcranial direct current stimulation.

**Figure 4.**
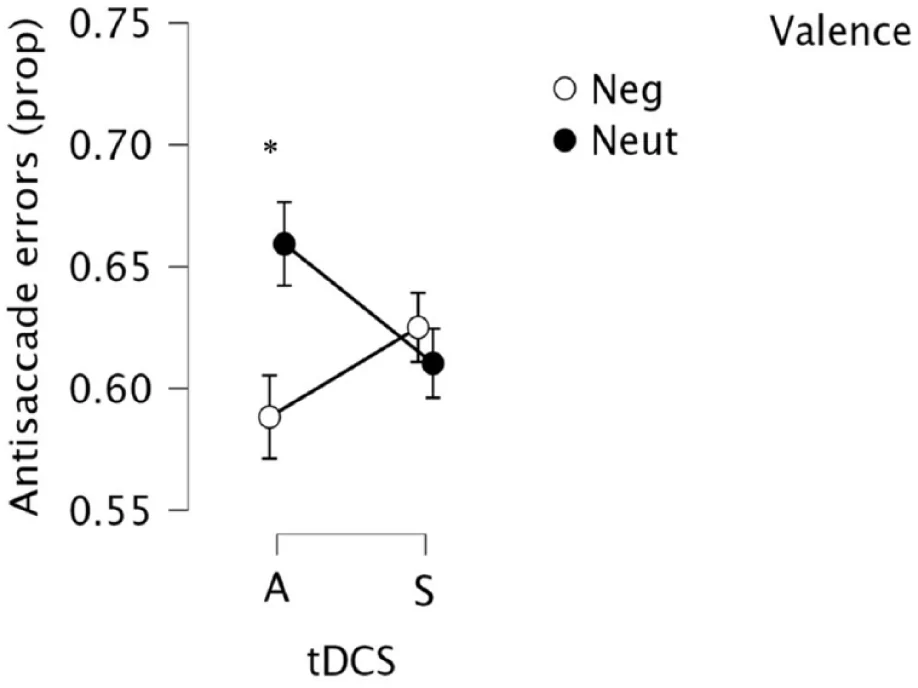
Eye-movement antisaccade errors toward negative and neutral stimuli during CO_2_ inhalation in “Active” and “Sham” groups (standard error bars). *p_Bonf_ = 0.013.

Consistent with previous studies, there was marked heterogeneity between the distributions of prosaccade error rates versus antisaccade error rates. Consequently, we ran additional separate group × valence analyses for prosaccades and antisaccades. Prosaccade accuracy was unaffected by group, valence and group × valence interaction (all *F*s < 1, *p*s > 0.49, *np*^2^s < 0.012). In contrast, antisaccade accuracy was moderated by valence × group (*F*(1,31) = 4.81, *p* = 0.036, *np*^2^ = 0.134), see Figure 4. Following active tDCS, participants made fewer erroneous eye movements to negative images (versus neutral images) (*t*(17) = 2.064, *p* = 0.05, *Cohen’s d point estimate* = 0.51)—there was no difference in the sham group (*t* < 1). This suggests that a single session of 20 minutes of active anodal tDCS to the left DLPFC may reduce erroneous orienting to threat during experimentally induced anxiety. Group differences were not moderated by baseline/change in mood, alertness, HR or blood pressure.

#### Latency

An omnibus group(2) × trial-type(2) × valence (2) ANCOVA revealed a main effect of trial type (*F*(1,34) = 83.6, *p* < 0.001), characterized by faster latencies for pro (*m* = 169 ms) as compared to antisaccade trials (*m* = 284 ms; see Table 4). We found no other significant main effects or interactions (*F*s < 1.4; *p*s > 0.25). Separate analyses of prosaccades and antisaccades did not reveal effects of group, valence or their interaction. This suggests that a single session of 20 minutes of active anodal tDCS to the left DLPFC does not modify eye-movement latencies toward or away from threatening stimuli during experimentally induced anxiety.

**Table 4.** Mean eye-movement latencies by trial type and valence in the active and sham tDCS conditions.

Saccade type	Active tDCS		Sham tDCS	
	*M*	SD	*M*	SD
Prosaccade Negative	174.85	31.67	159.67	14.42
Prosaccade Neutral	172.78	23.26	167.94	15.26
Antisaccade Negative	287.96	49.43	274.28	49.43
Antisaccade Neutral	270.41	57.19	272.81	52.40

tDCS: transcranial direct current stimulation.

#### Blinding

Similar to previous reports of tDCS blinding (e.g., Miler et al., 2018), participants could retrospectively identify their stimulation condition (active 14/18 correct; sham 11/18 correct). A chi-square test of independence revealed that the groups differed significantly in their retro-expectancy (binary measure) (*X*^2^ (1, *N* = 36) = 5.60, *p* = 0.018), with the sham group less accurate in determining whether they received active or inactive stimulation.

## Discussion

Twenty minutes of 2 mA anodal tDCS of left DLPFC using a balanced montage in healthy individuals was followed by fewer erroneous eye movements on threat versus neutral trials, when compared to sham stimulation. Supplementary analyses suggested this effect was characterized by fewer erroneous eye movements to threat images on antisaccade trials (versus neutral trials) following active stimulation, but not following sham stimulation. Collectively, these results suggest that active anodal tDCS has the potential to modify threat processing during periods of anxiety.

These findings support evidence from other neuromodulation studies of the effects of tDCS on attention to emotional information, and evidence in healthy volunteers that neuromodulation of DLPFC with tDCS (relative to sham) can reduce vigilance to threat; modify attentional disengagement from both positive and negative faces in an eye-tracking attention task; and increase executive attention control (e.g., Sanchez et al., 2016; Ironside et al., 2016). Our results complement evidence that tDCS modulates amygdala fear signaling, increasing frontoparietal attentional control, and reducing attentional capture by threat in high trait anxious individuals (Ironside et al., 2019), and can reduce attentional bias in patients with SAD (Heeren et al., 2017). Taken together, these findings suggest that neuromodulation by bipolar tDCS may facilitate disengagement from threat across a range of attentional tasks.

The present study is the first to examine the effect of tDCS on antisaccade performance in anxiety. A recent tDCS study in patients with binge eating disorder using a food-modified antisaccade task revealed no effect of either 1 or 2 mA anodal tDCS to the right dlPFC on saccade performance (Max et al., 2021).

We did not find clear effects of tDCS of left PFC (in a balanced montage) on eye-movement latency on prosaccade nor antisaccade trials. These results contrast with those from recent studies of tDCS-stimulated right DLPFC (as opposed to left DLPFC), which found no effects on attentional engagement, but effects on delayed attentional disengagement from emotional faces in a healthy sample (Sanchez et al., 2016).

Consistent with previous 7.5% CO_2_ challenge studies, we observed a large effect of CO_2_ inhalation on subjective anxiety and autonomic arousal (relative to baseline and post-stimulation). The effect of CO_2_ on subjective anxiety, blood pressure and heart were unaffected by tDCS, suggesting that differences in performance on the antisaccade task occurred independent of changes in subjective mood and autonomic arousal (see Baker et al., 2010; Raimondo et al., 2012). The null effect of tDCS on subjective anxiety is consistent with small effects reported in meta-analyses in clinical anxiety (Qi et al., 2024). Instead, evidence to date suggests tDCS may produce early effects on cognitive biases that could precede quantifiable effects on self-reported symptoms following prolonged use (akin to neuropsychological models of antidepressant drug action (Harmer and Cowen, 2013). If so, tDCS may be better used in augmentation protocols (rather than alone) to target cognitive-behavioral therapeutic mechanisms of action (as in CBT).

Earlier neuromodulation studies suggest that both active and sham tDCS protocols can lead to non-specific stress responses characterized by changes in skin temperature and cortisol levels (Baker et al., 2010; Raimondo et al., 2012). In contrast, our findings suggest that tDCS did not increase arousal before rising during CO_2_.

Physiological and psychological responses to CO_2_ covary with increased functional connectivity between medial frontal cortex and brainstem (Graham et al., 2024) and this effect appears to be greater in panic versus healthy controls, with panic patients exhibiting both stronger subjective response to 7% challenge and brainstem reactivity (Goossens et al., 2014). In our study, generalized anxiety (two weeks prior to test) did not covary with response to CO_2_ challenge nor effect of tDCS. We did not measure panic symptoms and so could not test whether this moderated anxiety and attention during CO_2_ challenge, nor whether tDCS had larger effects in those with elevated panic. Previous studies implicate fronto-limbic mechanisms in response to CO_2_. Baseline prefrontal-amygdala connectivity (before challenge) predicts increases in anxiety and heart rate during 7.5% CO_2_ (Huneke et al., 2020), and increased functional connectivity between limbic and anxiety regions during CO_2_ covaries with reported anxiety (Graham et al., 2024). The insula appears to be particularly important—increased subjective discomfort during CO_2_ correlates with increased insula activity (Goossens et al., 2014), and seed-based analyses suggest the insula as a hub for CO_2_-induced changes in connectivity to regions including the brainstem and amygdala (Graham et al., 2024). Consequently the insula interoceptive neural networks may be an important target for modulating the coupling of physiological response (e.g., altered breathing) and subjective anxiety response to CO_2._ We have previously provided some evidence that psychological exercises during the CO_2_ inhalation reduce anxiety in those who still experience increased physiological response (Ainsworth et al., 2015), and future studies may explore whether recent protocols that target insula with tDCS, for example (Esmaeilzadeh Kiabani et al., 2023) may reduce anxiety during physiological hyperarousal.

Limitations: Participant gender moderated the effect of CO_2_, with female participants exhibiting a stronger response to CO_2_. Gender effects in experimental medicine models such as CO_2_ are not well-characterized, though hormonal changes (e.g., during the menstrual cycle) can alter emotion processing and should be measured where possible. We did not include an active control site or low current control condition to test for dose response, and participants were able (when asked) to retrospectively discriminate the stimulation condition. The lack of an off-target active control condition is a limitation, particularly if unsuccessful blinding increases demand characteristics (e.g. increased motivation to perform well). In our study, though demand characteristics cannot be excluded, participants in the active condition did not display any improvements in mood or alertness, suggesting the effect of tDCS on the antisaccade task occurred independently of mood and motivation.

Previous studies suggest that tDCS can impact cognition via repeated exposure and, possibly, overnight consolidation, for example (Marshall et al., 2011)—consequently, future research should examine whether the effect of tDCS on DLPFC on attentional control is enhanced during repeated administration. Moreover, in the current study, participants received tDCS prior to (not during) 7.5% CO_2_ challenge—consequently, the concomitant effects of tDCS during anxiety provocation should be explored in future studies. Taken together, our findings provide a novel first test of tDCS in the CO_2_ experimental medicine model of anxiety. Initial evidence that tDCS may reduce attention to threat distractors during anxiety warrants follow-up in larger active-controlled studies.

## Notes

1. Though electrodes were centered over F3 and F4 to maximize stimulation of DLPFC we acknowledge that stimulation likely extended beyond these sites to broader PFC.
